# Aqueous Binder Enhanced High-Performance GeP_5_ Anode for Lithium-Ion Batteries

**DOI:** 10.3389/fchem.2018.00021

**Published:** 2018-02-12

**Authors:** Jun He, Yaqing Wei, Lintong Hu, Huiqiao Li, Tianyou Zhai

**Affiliations:** ^1^State Key Laboratory of Material Processing and Die and Mould Technology, School of Materials Science and Engineering, Huazhong University of Science and Technology, Wuhan, China; ^2^Shenzhen Research Institute of Huazhong University of Science and Technology, Shenzhen, China

**Keywords:** aqueous binder, alloy-type anode, GeP_5_, LiPAA, NaCMC, PVDF

## Abstract

GeP_5_ is a recently reported new anode material for lithium ion batteries (LIBs), it holds a large theoretical capacity about 2300 mAh g^−1^, and a high rate capability due to its bi-active components and superior conductivity. However, it undergoes a large volume change during its electrochemical alloying and de-alloying with Li, a suitable binder is necessary to stable the electrode integrity for improving cycle performance. In this work, we tried to apply aqueous binders LiPAA and NaCMC to GeP_5_ anode, and compared the difference in electrochemical performance between them and traditional binder PVDF. As can be seen from the test result, GeP_5_ can keep stable in both common organic solvents and proton solvents such as water and alcohol solvents, it meets the application requirements of aqueous binders. The electrochemistry results show that the use of LiPAA binder can significantly improve the initial Coulombic efficiency, reversible capacity, and cyclability of GeP_5_ anode as compared to the electrodes based on NaCMC and PVDF binders. The enhanced electrochemical performance of GeP_5_ electrode with LiPAA binder can be ascribed to the unique high strength long chain polymer structure of LiPAA, which also provide numerous uniform distributed carboxyl groups to form strong ester groups with active materials and copper current collector. Benefit from that, the GeP_5_ electrode with LiPAA can also exhibit excellent rate capability, and even at low temperature, it still shows attractive electrochemical performance.

## Introduction

Lithium-ion batteries (LIBs) is an important energy storage device, and anode material is one of the key factors to determine its comprehensive performance (Armand and Tarascon, 2008; Goodenough and Park, 2013). For the moment, graphite is the most widely used anode material in commercial LIBs due to its high reversibility and low potential. But the theoretical specific capacity of graphite is limited to 372 mAh g^–1^, which is too low to meet the increasing actual demand (Tarascon and Armand, 2001; Bruce et al., 2008). With the rapid development of electric vehicles, it is necessary to develop new high capacity anode materials to replace graphite (Guo et al., 2017). Alloy-type anode materials seem to be good choices as they can produce multiple electron reactions with lithium, as a result, they can provide much higher theoretical capacities than graphite (Xie et al., 2015b,c; Jiang et al., 2017). For example, the Si anode can exhibit an ultrahigh theoretical capacity of 4200 mAh g^–1^ by the formation of Li_4.4_Si alloy (Ma et al., 2016). However, the high capacity is always accompanied by huge volume changes, which would lead to a poor reversibility and cycle stability (Zhou et al., 2016). For example, the actual obtained reversible capacity of micro-Si (10 μm particles) is only about 1170 mAh g^–1^ with a low initial Coulombic efficiency of 35%, which is far below the theoretical value, and more seriously, 70% of its capacity drops only after five cycles (McDowell et al., 2012; Wu and Cui, 2012). Such a rapid capacity decay is due to the peeling-off and conductivity loss of active materials during pulverization by repeated large volume changes. In order to solve this problem, extensive methods have been introduced to promote the performance, such as reducing the particle size, designing nanostructured electrodes, or compositing active materials with carbon, to buffer the volume expansion/contraction upon lithiation/de-lithiation (Bruce et al., 2008; Jiang et al., 2012, 2016; He et al., 2014; Xie et al., 2015a; Yuan et al., 2016; Qin et al., 2017; Wang et al., 2017; Wei et al., 2017). Moreover, it is found that binder would play an important role especially for alloy-type anodes in maintaining the integrity of electrode, alleviating the stress caused by volume expansion, and keeping good contact between active substance and collector, therefore, the electrochemical performance can be greatly improved by using the proper binders (Chou et al., 2014; Yuca et al., 2014; Yue et al., 2014; Zhang et al., 2014; Liu et al., 2015). For example, Kovalenko et al. used an aqueous binder alginate for Si anode, and the cycle stability is much enhanced when compared to the PVDF system (Kovalenko et al., 2011).

GeP_5_ is a recently reported new alloy-type anode material for LIBs, it can deliver a large specific capacity of 2266 mAh g^–1^, which is much higher than graphite. And in comparison with Si, GeP_5_ shows a much higher reversibility upon lithiation/de-lithiation. Its obtained actual capacity is very close to the theoretical value (2289 mAh g^–1^). Besides, GeP_5_ holds a competitive conductivity to graphite, which is much higher than that of Si, thus it is expected to have high rate capability (Li et al., 2015, 2017). These promising properties suggest that GeP_5_ can be an excellent anode candidate for LIBs. However, similar to Si, GeP_5_ electrode also experiences volume expansion/contraction during discharge/charge processes upon alloying/de-alloying with lithium. Considering the importance of binder for Si anode, it is very possibly that a proper binder is highly needed for GeP_5_ to relief this problem. Compositing GeP_5_ with conductive carbon in nanoscale has been proved an effect way to relief this problem. However, as a new proposed anode material, the physical and chemical properties of GeP_5_ are not yet clear and there is no research on the influence of binder on its electrochemical performance.

Nowadays, poly (vinylidene fluoride) (PVDF) is the most commonly used binder in lithium-ion batteries because of its excellent electrochemical stability, good bonding capability, high adhesion, and universality. However, PVDF is very sensitive to the moisture. It is easy to deliquescence in moist air and lose the bonding ability. Besides, PVDF is usually used by employing N-Methyl pyrrolidone (NMP) as the solvent and dispersant, which is volatile, flammable, explosive, and high-toxic, leading to serious environment pollution. What's more, both PVDF and NMP are expensive, which lead to higher production costs of lithium-ion batteries. Based on these, the aqueous binders have drawn more and more attention in recent years because of the advantages of low cost and environmental friendly (Zhang et al., 2004). It has been reported that some aqueous binders can bring better electrochemical performance than PVDF to some anode and cathode materials (Cai et al., 2009; Courtel et al., 2011; Kovalenko et al., 2011; Chai et al., 2013; Gong et al., 2013; Kuruba et al., 2015; Liu et al., 2017), especially for Si and Sn anode (Hochgatterer et al., 2008; Bridel et al., 2010; Koo et al., 2012; Erk et al., 2013; Yim et al., 2013; Chou et al., 2014; Pieczonka et al., 2015). These binders may be also good choices for alloy-type GeP_5_ anode. So, in this manuscript, we systematically study the electrochemical properties of GeP_5_ electrode in both aqueous and non-aqueous binder systems. Two most used aqueous binders, lithium-polyacrylic acid (LiPAA), and sodium-carboxymethylcellulose (NaCMC), are chosen to investigate the effects of binder on the performances of GeP_5_ in comparison with the traditional non-aqueous PVDF binder. Through the result of the stability test, it found that GeP_5_ can keep stable in the protonic solvent, therefore, it indicates that aqueous binders can be used for GeP_5_ anode. The electrochemistry results show that the use of LiPAA binder can significantly improve the initial Coulombic efficiency, reversible capacity, and cyclability of GeP_5_ anode. Even at a low temperature of −20°C, GeP_5_ can remain a large capacity of 1154 mAh g^–1^, further showing that GeP_5_ is a very promising candidate for high energy LIBs.

## Experimental

### Materials preparation and characterization

The pure phase GeP_5_ was synthesized via a high energy mechanical milling (HEMM, Fritsch Pulverisette-6) method (Li et al., 2015). Twenty millimoles Ge and One hundred millimoles red P powders were added into a stainless steel tank with a rotation speed of 400 rpm for 6 h under Ar atmosphere. GeP_5_/C nanocomposite was also synthesized by HEMM pure GeP_5_ and super P (in a mass ratio of 7:2) with a rotation speed of 400 rpm for 10 h under Ar atmosphere. The obtained products were characterized by X-ray diffraction (XRD, PANalyticalX'pert PRO-DY2198), confocal Raman spectrometer (Raman, jobinYvon HR800), scanning electron microscope (SEM, FEI Quanta650 FEG) and Fourier transform infrared spectroscopy (FT-IR, Bruker VERTEX 70).

### Electrochemical performance

For the electrochemical evaluation, the homogeneous slurry which was obtained by mixed GeP_5_/C powder with binder (LiPAA in deionized water, NaCMC in deionized water, and PVDF in NMP, respectively) in a weight ratio of 90:10 was coated on a copper foil substrate. As-coated electrodes were dried at 80°C on a vacuum oven for 12 h. The mass loading of the electrodes is ca. 1–1.5 mg cm^−2^. Two thousand thirty-two coin-type electrochemical cells were assembled as lithium metal as the counter and reference electrodes in an Ar-filled glove box with water-oxygen content below 1 ppm. 1.0 mol L^−1^ LiPF_6_ dissolved in ethylene carbonate (EC), dimethyl carbonate (DMC) and ethyl methyl carbonate (EMC) (EC: DMC: EMC = 1:1:1vol. %) solution was the electrolyte. All the cells were tested galvanostatically between 0.005 V and 3.0 V (vs. Li/Li^+^) at different current densities (100–2000 mA g^−1^) using LAND (Wuhan Kingnuo Electronic Co, China) tester. The capacity was calculated based on the active mass of the electrodes. DQ/dV was performed at a voltage range of 0.005–3.0 V by using the automatic battery testing system (Hokuto Denko, HJ1001SD8).

## Results and discussion

**Figure 1:**
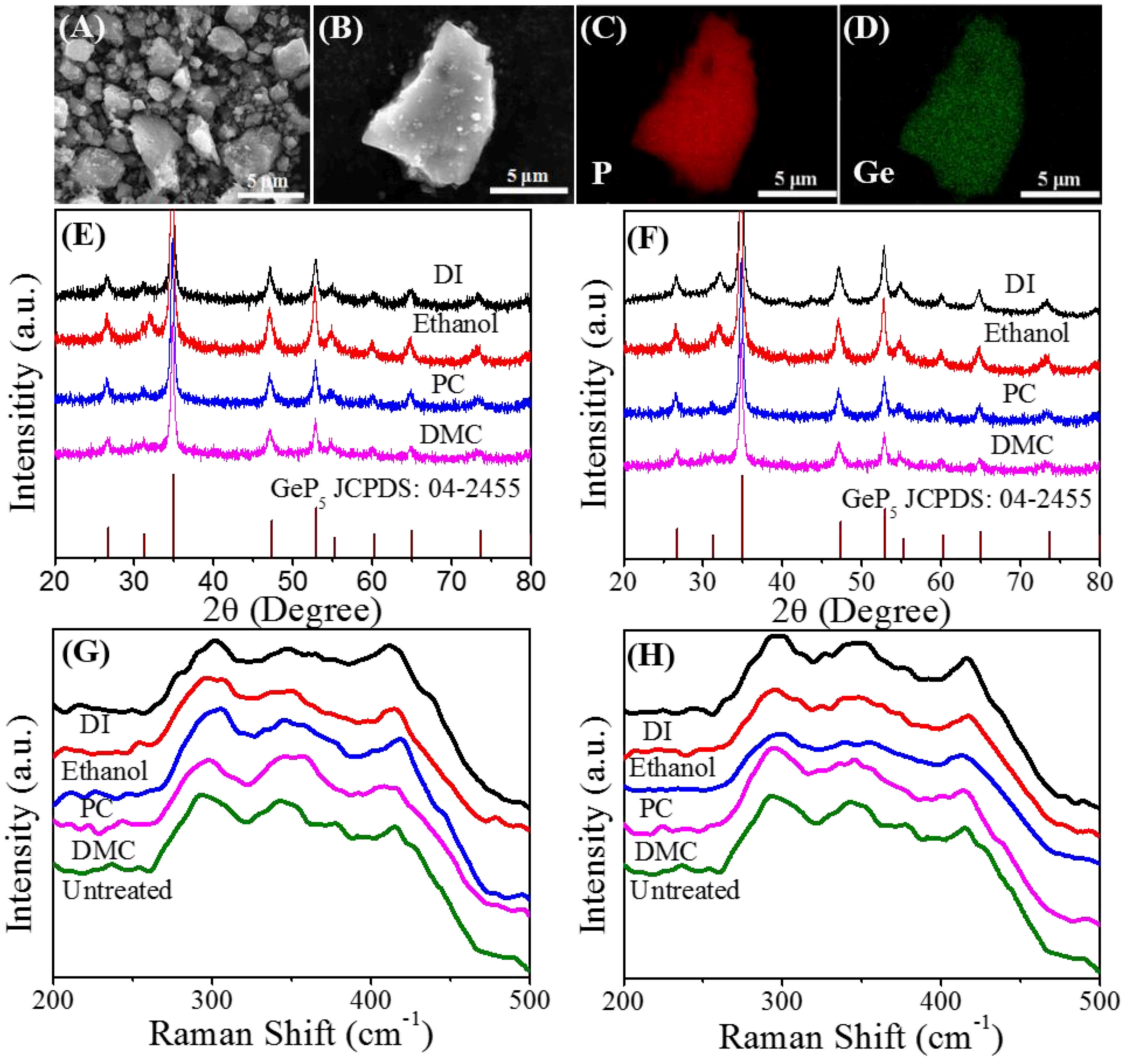
**(A,B)** The SEM images and **(C,D)** the mapping images of GeP_5._ The XRD and Raman patterns of GeP_5_ after 96 h in different kinds of solvent at **(E,G)** room temperature, **(F,H)** 50°C.

The GeP_5_ synthesized via HEMM are stacked by irregular micron particles, and the mapping images show that element Ge and P are uniformly distributed in the GeP_5_ particle (Figures 1A–D). As a newly reported anode material, the physical, and chemical properties of GeP_5_ are not yet very clear. Before introducing different binder, the chemical and physical stability of GeP_5_ in different solvents should be verified. For aqueous binder, protic solvent is usually used, while for non-aqueous binder, NMP is mostly used. Besides, the organic carbonate solvents in electrolyte are also directly contact with electrode materials. So we first test that whether GeP_5_ can stay stable and won't dissolve or undergo chemical reactions in the different kinds of binder dispersants and electrolyte solvents. In the test, deionized water (DI), propylene carbonate (PC), dimethyl carbonate (DMC) and ethanol are selected as the solvents, and 500 mg GeP_5_ powder was immersed in the above solvent for 96 h at room temperature, respectively. In addition, in order to further accelerate the reaction, the same test is carried out at 50°C. Figures 1E,F show the XRD patterns of the GeP_5_ powder after soaking treatment. It can be found that the diffraction peaks of all the samples are complete coincide with GeP_5_ (JCPDS Card No. 04-2455). And the Raman patterns of the samples are showed in Figures 1G,H, there is also no difference between the treated and untreated GeP_5_ powder. The results indicate that GeP_5_ does not undergo chemical reactions with the above solvents no matter at room temperature or at 50°C, showing good chemical stability of GeP_5_ in both aprotic and protic solvents. It promises the utilization possibility of aqueous binders in GeP_5_ anode.

**Figure 2:**
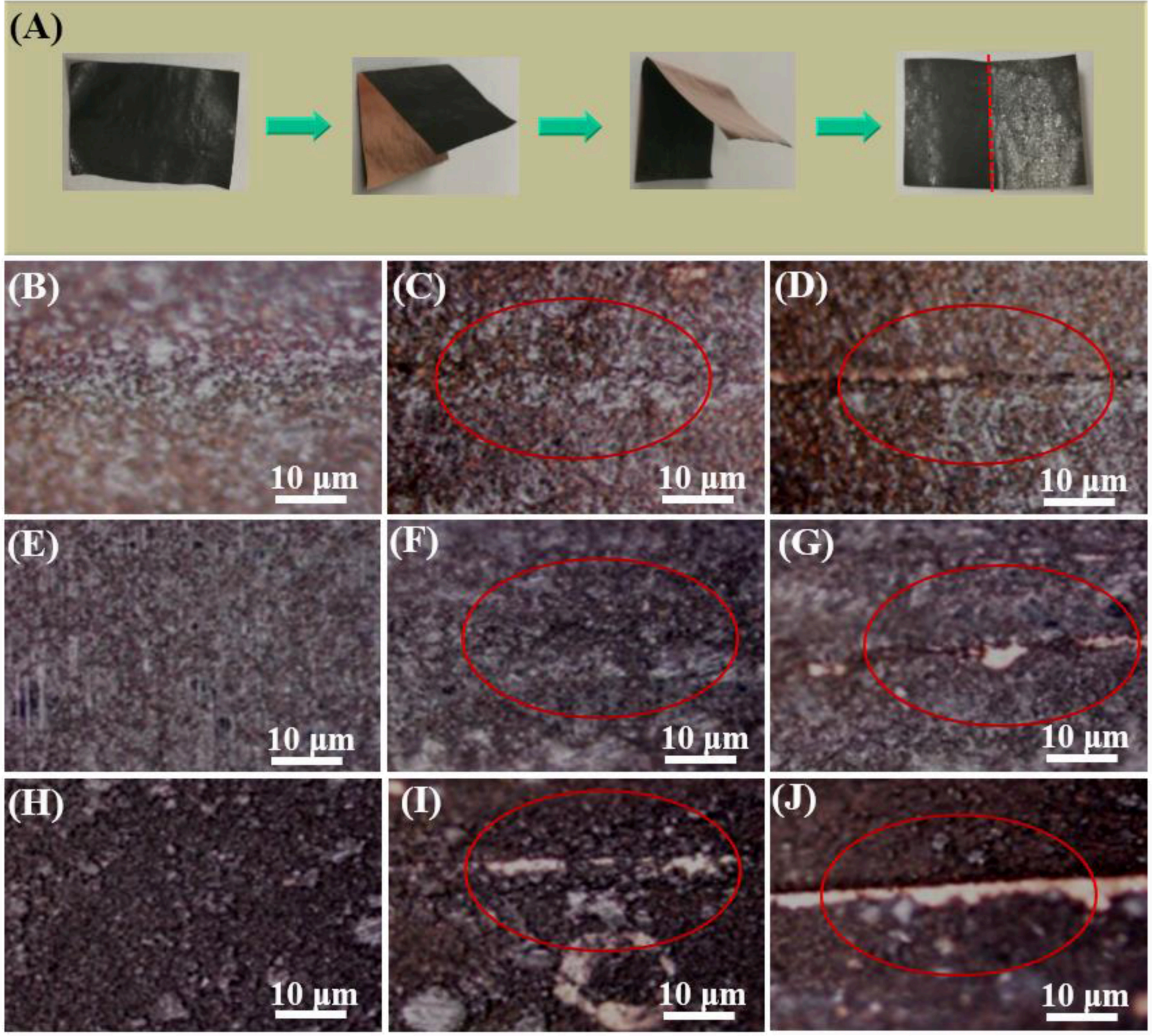
**(A)** The schematic diagram of folding electrodes; The microscope photos of GeP_5_ electrodes with **(B)** LiPAA before being folded, **(C)** LiPAA after being folded five times, **(D)** LiPAA after being folded 10 times; **(E)** PVDF before being folded, **(F)** PVDF after being folded five times, **(G)** PVDF after being folded 10 times; **(H)** NaCMC before being folded, **(I)** NaCMC after being folded five times, **(J)** NaCMC after being folded 10 times.

To test the adhesion capability of LiPAA, NaCMC, and PVDF, the different binder-based electrodes were successively folded for different times as Figure 2A shown. The microscope photos are shown in Figures 2B–J. Compared with the unfolded electrodes, it can be found that after been folded for five times, the structural integrity of LiPAA-based binder electrode and PVDF-based binder electrode were kept well, however, there were cracks appeared at the NaCMC-based electrode, and some active material exfoliated from the copper current collector. And when the fold times increased to 10, the surface morphology of NaCMC-based electrode getting worse, the cracks became more apparent, and more active material was peeling off. In comparison, the PVDF-based electrode is much better than the NaCMC-based one. There was only a small amount of active material exfoliated. The best adhesion capability can be seen from the LiPAA-based electrode, after ten-time-folded, there was no active material exfoliate from the copper current collector. According to the test, it is clearly that LiPAA have the best adhesion capability among the three kinds of binders.

**Figure 3:**
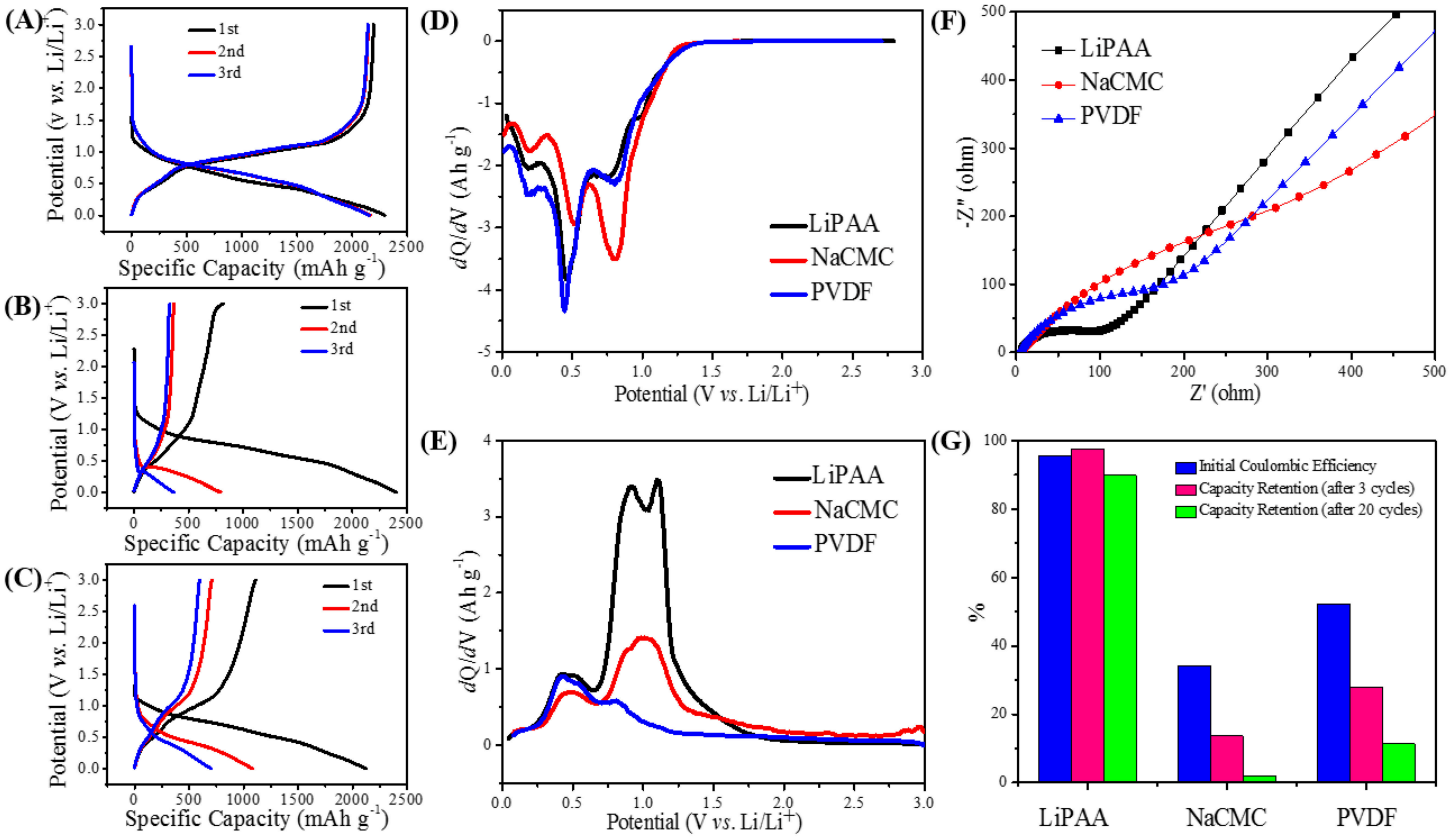
The cycle curves in the first three cycles of GeP_5_ electrodes with **(A)** LiPAA, **(B)** NaCMC, **(C)** PVDF. The dQ/dV curves of GeP_5_ electrodes **(D)** in first discharge, **(E)** in first charge. **(F)** Cell impedance of GeP_5_ electrodes after thirty cycles. **(G)** The electrochemical performance comparison of CeP_5_ electrodes with different binders.

The discharge/charge profiles of GeP_5_ electrode with LiPAA, PVDF, and NaCMC binder are presented in Figures 3A–C, respectively. The LiPAA system shows the best cycle performance, at a current density of 100 mA g^–1^, it delivers discharge and charge capacities of 2294 and 2193 mAh g^–1^ at the first cycle. After 3 cycles, there was only a slight drop in capacity. In contrast, the NaCMC system exhibits a first discharge/charge capacity of 2406/818 mAh g^–1^, and after 3 cycles, the reversible capacity was decayed to less than 400 mAh g^–1^. As for PVDF system, a discharge capacity of 2122 mAh g^–1^ and a charge capacity of 1108 mAh g^–1^ were obtained at the first cycle, and they decreased to 700 and 594 mAh g^–1^ at the third cycle, respectively. It is a little better than the NaCMC one, but still far worse than the LiPAA system.

In order to understand the details of charge discharge data more clearly, dQ/dV profiles of the GeP_5_ electrodes in the first cycle with LiPAA binder, NaCMC binder, and PVDF binder are shown in Figures 3D,E. At the first discharge (Figure 3D), all of the three systems exhibit two distinct peaks around 0.5 and 0.8 V, respectively, which are related to the forming of conversion reaction product Li_x_P, and a small peak around 0.18 V which is attributed to the forming of alloying product Li_x_Ge. According to that, GeP_5_ electrodes with all the three binders can be fully lithiated at the first discharge process. As for the first charge, the peak around 0.5 V, which assigned to the extraction of lithium from Li_x_Ge, can be found clearly in all the three binder systems, it means that de-alloying process can be fully completed. However, the peaks around 1.0 V that correspond to the extraction of lithium from Li_x_P are much difference among them. The LiPAA system shows the much more obvious redox peak than the other two binder systems. It indicates the highest reversibility of lithiation/de-lithiation of GeP_5_ in LiPAA system, which is consistent well with the discharge/charge curves. As a summary, the initial Coulombic efficiency and capacity retention of the three binder systems are listed in Figure 3G, it can be clearly observed that LiPAA system has much higher initial Coulombic efficiency of 95.63% than the NaCMC system of 34.03% and the PVDF system of 52.21%. And it also shows the highest capacity retention than the other two binder systems. There are 97.62, 13.63, and 28.02% of the capacity retained after 3 cycles for the LiPAA system, NaCMC system, and PVDF system, respectively. And when the cycle number increased to 20, the capacity retention values are reduced to 89.79, 2.02, and 11.33% for the three systems, respectively.

The electrochemical impedance spectroscopy (EIS) measurements of GeP_5_ electrodes with different binders were also conducted. The Nyquist plots of GeP_5_ electrodes with different binders after 30 cycles were shown in Figure 3F. It's obvious that the impedance spectrum consist of a depressed semicircle in the high frequency region and a straight line in the low frequency region. The former is related to the charge transfer resistance, while the latter is attributed to the diffusion of Li^+^ within the bulk of active materials, which called the Warburg resistance. According to the impedance spectra data, it can be found that after 30 cycles, the GeP_5_ electrode with LiPAA binder shows smaller charge transfer resistance in the high frequency region than those of PVDF and NaCMC binder systems. And the result indicates that the LiPAA system shows the favorable kinetics of electrode reactions.

**Figure 4:**
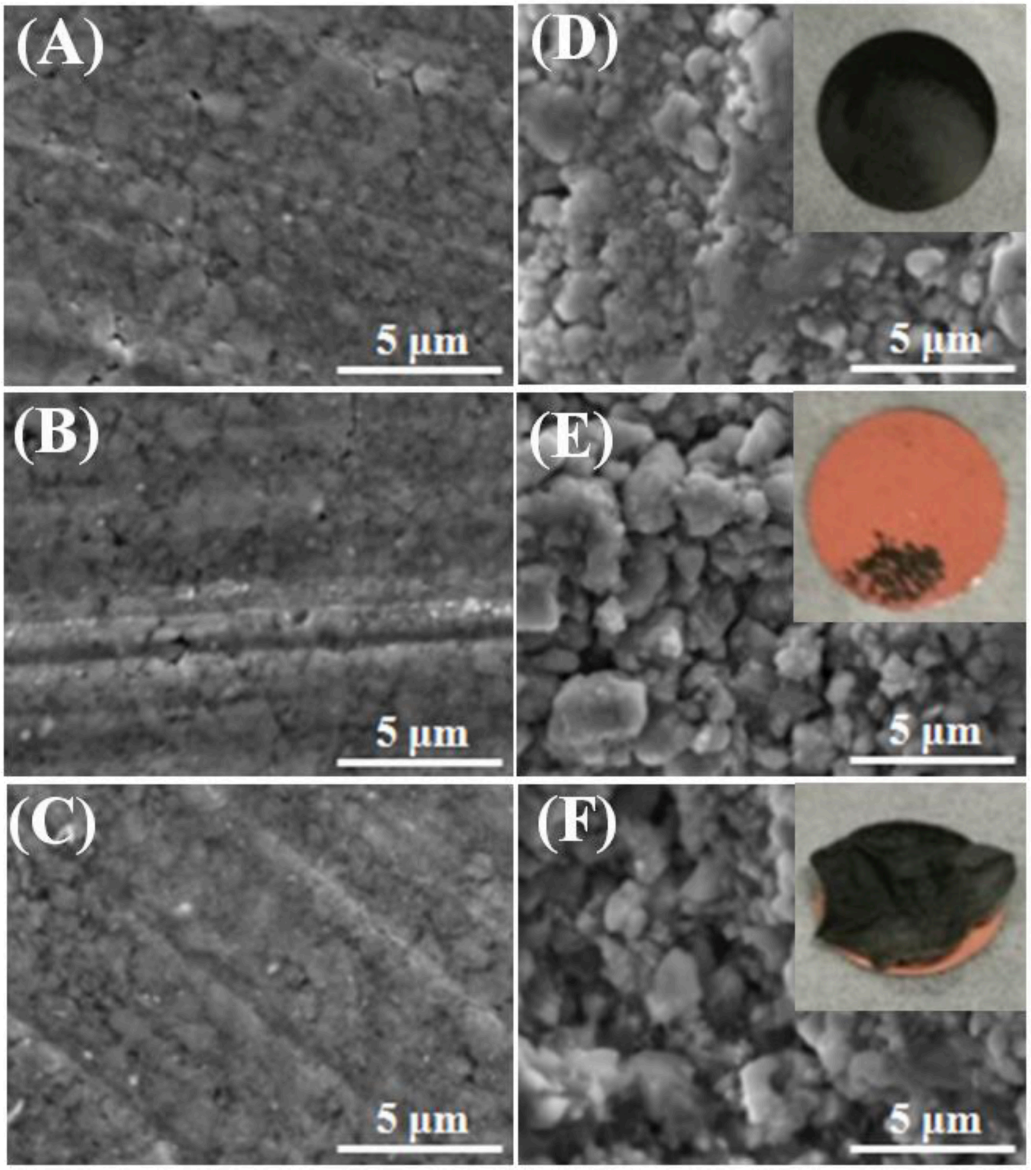
SEM images of GeP_5_ electrodes with **(A)** LiPAA before cycling, **(D)** LiPAA after one cycle and photo of GeP_5_ electrode with LiPAA after thirty cycles (inset); **(B)** NaCMC before cycling, **(E)** NaCMC after one cycle and photo of GeP_5_ electrode with NaCMC after thirty cycles (inset); **(C)** PVDF before cycling, **(F)** PVDF after one cycle and photo of GeP_5_ electrode with PVDF after thirty cycles (inset).

To further verify the difference in cycle stability, the electrodes were extracted from the coin cells after 30 cycles, and compared with the fresh one. The morphologies of the GeP_5_ electrodes with three binders were observed by SEM, and the surface conditions of them are shown in Figure 4. The electrode conditions of the three systems are almost same before cycle test (Figures 4A–C), however, there are much difference between them in the morphology after cycle life. The morphology of the LiPAA-based electrode was kept well after one cycle (Figure 4D). It still exhibited a smooth surface, and the active material firmly adhered to the copper substrate even after 30 cycles (inset photo). As for the other two systems, both of the two electrodes showed rough surfaces after one cycle life (Figures 4E,F), and what's more, the active material was severely pulverized and peeled off from the copper substrate after 30 cycles (inset photo), as a result, the reversible capacity faded rapidly. What's more, the change of electrode surface morphology is regarded as the main reason for the high contact resistance between active materials and copper collector.

**Figure 5:**
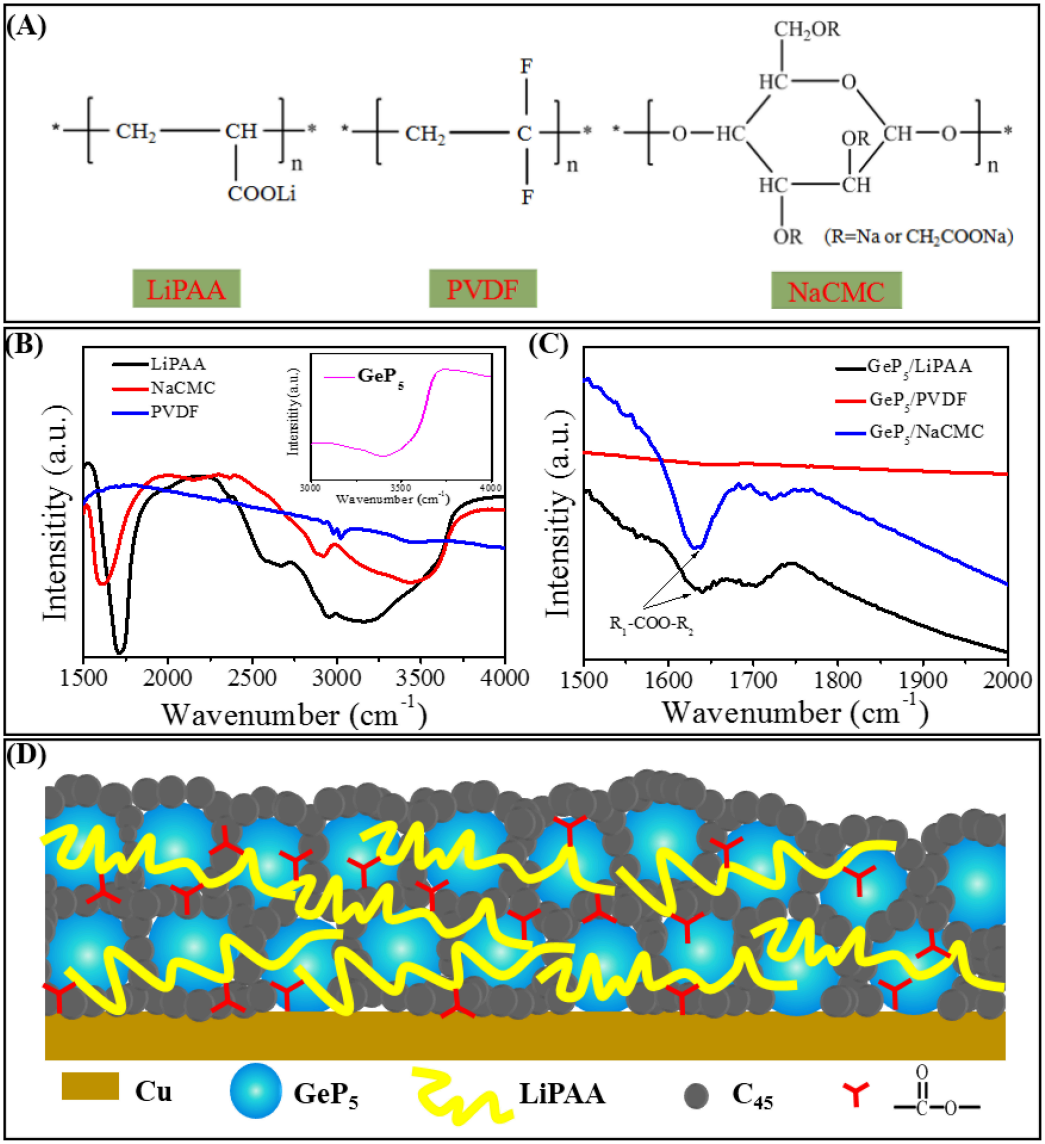
**(A)** The molecular structural formulas of LiPAA, PVDF, NaCMC. The FT-IR spectrum of **(B)** LiPAA, NaCMC, PVDF, and GeP_5_ (inset), **(C)** LiPAA/GeP_5_, NaCMC/GeP_5_, PVDF/GeP_5_. **(D)** The adhesive mechanism schematic illustration of LiPAA binder in GeP_5_ electrode.

According to the above description, LiPAA binder system shows much better electrochemical performance than NaCMC and PVDF systems, and it mainly owning to the difference between their molecular structures. As shown in Figure 5A, both LiPAA and PVDF exhibit long-chain macromolecule structures, however, NaCMC displays a ring polymer structure. By comparison, the long-chain structure seems has a better adhesive property than the ring structure. PAA shows a breaking stress value (σ_b_) about 90 MPa, which is much higher than CMC (30 MPa), and PVDF (37 MPa) (Magasinski et al., 2010). Besides, PAA also shows much stronger resistance to deformations than PVDF, which makes it can effectively resist stress changes caused by volume expansion and keep active materials contacting well with copper collector (Fan et al., 2001; Antonova, 2009). In contrast, the weak resistance of PVDF to deformations results in poor cycle stability in alloy-type anodes which with huge volume change upon the discharge/charge process. In addition, there are large numbers of carboxyl groups that uniformly distributed in the molecular chain of LiPAA. And as the diagram indicating (Figure 5D), these carboxyl groups can bonding with hydroxyl groups that exist on the surface of active materials, conductive agents and copper substrate, as a result, it greatly strengthens the adhesive capacity of LiPAA binder. Benefit from that, LiPAA can better resist the stress change caused by huge volume expansion/contraction during discharge/charge process. The active materials can maintain good contact with each other and keep good adhesion to the current collector during the cycle life. As a result, the integrity of the electrode structure can be well maintained, which ensures enhanced cycle stability. For PVDF, there is no carboxyl groups or hydroxyl groups exist, and its adhesion effect is only depends on intermolecular forces (Van der Waals' force). As Figure 5B shows, the FT-IR spectrum further verify the existence of hydroxyl groups and carboxyl groups in LiPAA and NaCMC, which are correspond to the peaks that located around 2500–3500 cm^–1^ and 1700 cm^–1^, respectively, it also indicates that there are oxygen-containing functional groups (3200–3700 cm^–1^) in GeP_5_, which can bonding with LiPAA and NaCMC. However, there is no peak at these positions can be found for the PVDF binder. After mixed with GeP_5_, the FT-IR spectrum clearly display a peak located around 1630 cm-1 in both curves of GeP_5_ with LiPAA and NaCMC, which is characteristic for the ester R_1_-COO–R_2_ bond. As compared, there are not any peaks here on the curve of GeP_5_ with PVDF binder (Figure 5C).

**Figure 6:**
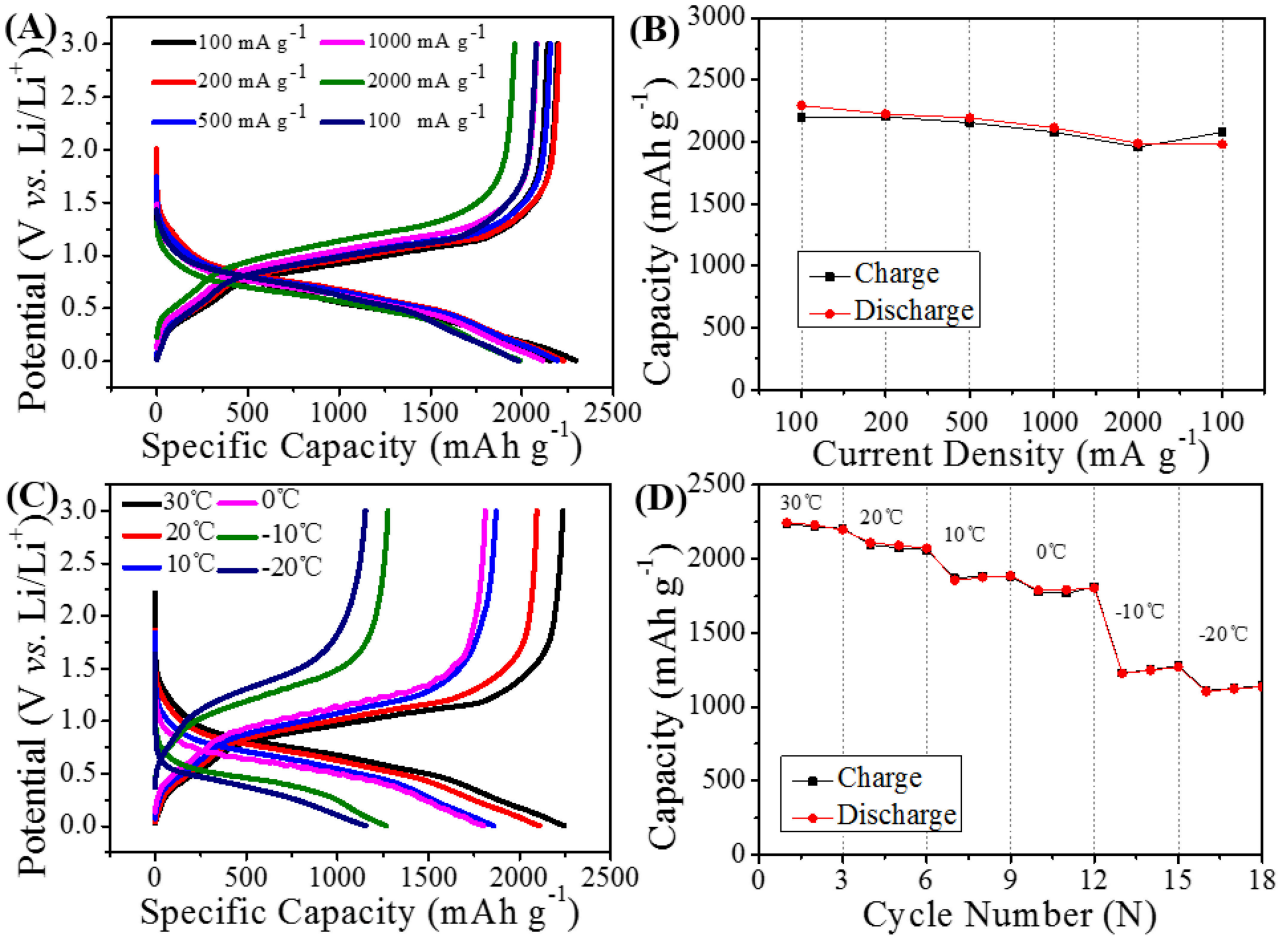
**(A)** Cycle curves and **(B)** rate performance of GeP_5_ electrode with LiPAA binder in different current density. **(C)** Cycle curves in different temperature, and **(D)** temperature capability of the GeP_5_ electrode with LiPAA binder.

According to the above results, the water-based LiPAA binder can exhibit much better cycle performance than the traditional PVDF binder and another water-based NaCMC binder in GeP_5_ electrode. Further electrochemical test is conducted to investigate the bonding properties of LiPAA at different working conditions. The rate capability of GeP_5_ electrode with LiPAA binder was shown in Figures 6A,B. The half-cell was cycled at the current density of 100, 200, 500, 1000, and 2000 mA g^–1^ and back to 100 mA g^–1^ in succession. As can be seen from the Figure 6A, the charging and discharging platforms are well maintained with slight change in capacity. It indicates that the discharge-charge reactions can be relatively completed even at a high current density. A discharge capacity of 2294, 2203, 2194, 2115, and 1988 mAh g^–1^ can be obtained at 100, 200, 500, 1000, and 2000 mA g^–1^, respectively. And there are about 86.3% capacity retained in the current of 2000 mA g^–1^ when compared with the current density of 100 mA g^–1^. When the current density came back to 100 mA g^–1^, the capacity can back to a high level. The attractive rate performance indicates LiPAA binder can still hold good bonding effect even at high current density.

Binder as a polymer material, its physical properties would change with the change of temperature. Up to now, most of the research about alloy-type anode materials is based on room temperature conditions, and there are little reports on their electrochemical performance at low temperature. As GeP_5_ electrode with LiPAA binder shows excellent cycle stability and rate capability at room temperature, we change the battery test temperature to verify whether the good electrochemical performance can be maintained in low temperature environment. A temperature range from 30 to −20°C was selected for testing of GeP_5_ electrode with LiPAA binder at 200 mA g^−1^, and the result was shown in Figures 6C,D. It can be found that the reversible capacity decreases gradually with the drop of temperature, and the voltage polarization is also becoming more and more obvious. And this is attributed to the suppressed reaction dynamic at low temperature. A discharge capacity of 2245, 2094, 1856, 1796, and 1266 mAh g^−1^ can be exhibited at 30, 20, 10, 0, and −10°C, even at a low temperature of −20°C, the GeP_5_ electrode with LiPAA can still provide a high discharge capacity of 1154 mAh g^−1^. The result shows that LiPAA can keep good bonding property at low temperature.

## Conclusion

As compared with traditional PVDF, aqueous-based binder systems have many advantages, such as low cost, environment friendly, and non-toxic, and they have attracted much attention as replacers of PVDF. In this work, we tried to apply aqueous-based binders (LiPAA and NaCMC) to the promising alloy type anode material GeP_5_, which can provide a high theoretical capacity near to 2300 mAh g^–1^, and made a comparison with traditional PVDF binder. According to the result, the LiPAA system shows the best physical adhesion capability and electrochemical performance. The initial Coulombic efficiency and cycle stability are greatly improved when compared with NaCMC and PVDF. And the improvement is mainly ascribed to the unique molecular structure of LiPAA. First, it shows a high strength long chain polymer structure, which can effectively resist the stress change caused by volume expansion/contraction. Second, there are lots of carboxyl groups uniformly distributed on the molecular chain, which can react with hydroxyl groups that exist on the surface of active materials, conductive agents and copper substrate, and the adhesion ability can be improved. Benefit from that, the GeP_5_ electrode with LiPAA can exhibit excellent cycle stability and rate capability, and even at low temperature, it can also show attractive electrochemical performance. We believe that the aqueous-based binder LiPAA has a greater application prospect in the future.

## Author contributions

All authors listed have made a substantial, direct and intellectual contribution to the work, and approved it for publication.

### Conflict of interest statement

The authors declare that the research was conducted in the absence of any commercial or financial relationships that could be construed as a potential conflict of interest.
